# RETRACTED: Ali et al. A Novel Herbal Hydrogel Formulation of *Moringa oleifera* for Wound Healing. *Plants* 2021, *10*, 25

**DOI:** 10.3390/plants13111502

**Published:** 2024-05-30

**Authors:** Aaliya Ali, Prakrati Garg, Rohit Goyal, Gurjot Kaur, Xiangkai Li, Poonam Negi, Martin Valis, Kamil Kuca, Saurabh Kulshrestha

**Affiliations:** 1 Faculty of Applied Sciences and Biotechnology, Shoolini University of Biotechnology and Management Sciences, Bajhol, Solan 173229, Himachal Pradesh, India; aaliyaali78600@gmail.com (A.A.); prakrati417@gmail.com (P.G.); 2 Center for Omics and Biodiversity Research, Shoolini University of Biotechnology and Management Sciences, Bajhol, Solan 173229, Himachal Pradesh, India; 3 School of Pharmaceutical Sciences, Shoolini University of Biotechnology and Management Sciences, Solan 173229, Himachal Pradesh, India; rohitgoyal@shooliniuniversity.com (R.G.); gurjotkaur@shooliniuniversity.com (G.K.); poonamgarge@gmail.com (P.N.); 4 Ministry of Education Key Laboratory of Cell Activities and Stress Adaptation, School of Life Science, Lanzhou University, Tianshuinanlu #222, Lanzhou 730000, China; xkli@lzu.edu.cn; 5 Department of Neurology of the Medical Faculty of Charles University and University Hospital in Hradec Kralove, Sokolska 581, 50005 Hradec Kralove, Czech Republic; martin.valis@fnhk.cz; 6 Department of Chemistry, Faculty of Science, University of Hradec Kralove, 50005 Hradec Kralove, Czech Republic; 7 Biomedical Reseaerch Center, University Hospital Hradec Kralove, Sokolska 581, 50005 Hradec Kralove, Czech Republic

The journal retracts the article, “A Novel Herbal Hydrogel Formulation of *Moringa oleifera* for Wound Healing” [1], cited above. 

Following publication, concerns were brought to the attention of the publisher regarding overlapping figure panels within this article [1]. 

Adhering to our procedure, an investigation was conducted by the Editorial Office and Editorial Board that confirmed the overlap with Figure 2, specifically subfigure “5% Hydrogel—Day 13” and “5% Hydrogel—Day 13”. While the authors cooperated within the investigation, they were unable to satisfactorily explain the overlap nor verify the integrity of the abovementioned image panels. As a result, the Editorial Board has lost confidence in the integrity of the findings and decided to retract this publication [1], as per MDPI’s retraction policy (https://www.mdpi.com/ethics#_bookmark30) and in line with the Committee on Publication Ethics retraction guidelines (https://publicationethics.org/retraction-guidelines). 

This retraction was approved by the Editor-in-Chief of the journal *Plants*. 

The authors did not agree to this retraction. 
